# Depression Fully Mediates the Effect of Multimorbidity on Self-Rated Health for Economically Disadvantaged African American Men but Not Women

**DOI:** 10.3390/ijerph16101670

**Published:** 2019-05-14

**Authors:** Shervin Assari, James Smith, Mohsen Bazargan

**Affiliations:** 1 Department of Family Medicine, Charles R Drew University of Medicine and Science, Los Angeles, CA 90095, USA; jamessmith@cdrewu.edu (J.S.); mobazarg@cdrewu.edu (M.B.); 2 Department of Family Medicine, University of California Los Angeles (UCLA), Los Angeles, CA 90095, USA

**Keywords:** race, gender, Blacks, African Americans, ethnic groups, chronic medical conditions, depression, self-rated health

## Abstract

*Background*. Although chronic medical conditions (CMCs), depression, and self-rated health (SRH) are associated, their associations may depend on race, ethnicity, gender, and their intersections. In predominantly White samples, SRH is shown to better reflect the risk of mortality and multimorbidity for men than it is for women, which suggests that poor SRH among women may be caused not only by CMCs, but also by conditions like depression and social relations—a phenomenon known as “the sponge hypothesis.” However, little is known about gender differences in the links between multimorbidity, depression, and SRH among African Americans (AAs). *Objective*. To study whether depression differently mediates the association between multimorbidity and SRH for economically disadvantaged AA men and women. *Methods*. This survey was conducted in South Los Angeles between 2015 to 2018. A total number of 740 AA older adults (age ≥ 55 years) were enrolled in this study, of which 266 were AA men and 474 were AA women. The independent variable was the number of CMCs. The dependent variable was SRH. Age and socioeconomic status (educational attainment and marital status) were covariates. Depression was the mediator. Gender was the moderator. Structural Equation Modeling (SEM) was used to analyze the data. *Results*. In the pooled sample that included both genders, depression partially mediated the effect of multimorbidity on SRH. In gender specific models, depression fully mediated the effects of multimorbidity on SRH for AA men but not AA women. For AA women but not AA men, social isolation was associated with depression. *Conclusion*. Gender differences exist in the role of depression as an underlying mechanism behind the effect of multimorbidity on the SRH of economically disadvantaged AA older adults. For AA men, depression may be the reason people with multimorbidity report worse SRH. For AA women, depression is only one of the many reasons individuals with multiple CMCs report poor SRH. Prevention of depression may differently influence the SRH of low-income AA men and women with multimorbidity.

## 1. Introduction

Self-rated health (SRH) is a widely accepted indicator of overall health. Poor SRH predicts risk of mortality [1,2,3,4,5,6,7,8,9] in both community [10] and clinical [11] settings. For both the general population [10] and patients with a chronic disease [12], SRH reflects long-term risk of mortality. SRH is a standard outcome in randomized clinical trials (RCTs) [13,14,15,16] and in national cohort surveys in Europe [17,18,19] as well as the US [20,21]. In the US, the Health and Retirement Study (HRS) [22], the Panel Study of Income Dynamics (PSID) [20,21], and the National Health and Nutrition Examination Survey (NHANES) [10] all measure health at the population level using SRH. SRH is also used for cross-country comparisons [23,24,25,26,27,28,29] and policy development [30,31,32,33]. It is used as a reflection of health disparities and inequality [34,35,36,37]. SRH is also used to track the subjective health of individuals with index psychiatric or medical conditions [38].

Although SRH is known to be a valid health measure [1,2,3,4,5,6,7,8,9], SRH may not reflect the same aspects of health across populations distinguished by race, ethnicity, and gender [39]. Although SRH is efficient, cost effective, and time saving [40], poor SRH may not have the same meaning for men as for women [41]. Despite the high acceptability of SRH as a measure of health [1,2,3,4,5,6,7,8,9], SRH may mean different things for different populations.

The use of SRH for group comparisons may be questioned if it is not universally valid and comparable across racial [39] and gender lines [41]. Age, gender, socioeconomic status (SES), health behaviors, chronic medical conditions (CMCs), and depression may differently influence the SRH of people in different countries [42]. If poor SRH means different things for subsections of populations, any comparison of population groups using SRH would be biased [41,43]. Thus, SRH would not be the ideal tool for measuring health in diverse populations [43,44].

Although not all studies agree [45], a large body of evidence suggests that poor SRH may not reflect the same health for subpopulations classified by age, gender, ethnicity, and health status [45,46,47,48,49,50,51,52]. For example, the meaning of SRH may shift according to developmental stage and age [45,47]. Race and ethnicity alter what poor SRH reflects [53,54,55,56]. This is in part because the reference group of each section of the population differs [57,58,59,60,61,62]. Similarly, non-health determinants of SRH vary by race and gender [63,64,65]. For example, socioeconomic status [42,43,63,65] and neighborhood [66] differently impact the SRH of racial and gender groups. In addition, the role of physical health in shaping SRH is not constant across various populations [42]. Finally, even within a given patient population, SRH differently reflects the severity of the condition and outcomes in different racial and gender groups [1,67].

Differences in what shapes SRH may result in differences in the validity of SRH as a predictor of the risk of mortality in different groups [41,56]. Thus, while poor SRH may be an excellent marker of mortality risk for White men, it may not be for African Americans (AAs), Hispanics, or even women [42,43,68]. To understand whether cross-gender, cross-racial, and cross-ethnic comparisons of SRH are valid, we need to compare determinants of poor SRH across various groups. Cross-group comparisons of SRH will only be valid if SRH has the same meaning across populations.

### Aims

To better understand how gender impacts SRH in AA older adults [54,69,70,71], this study compared the mediating effect of depression on the effect of multimorbidity on SRH between AA men and AA women. In line with the sponge hypothesis [41,67], we expected multimorbidity to have a stronger effect on SRH among AA men than among AA women. We also expected depression to have a stronger effect on SRH among AA women than among AA men. The sponge hypothesis suggests that, for women, SRH is more inclusive, acting like a sponge to absorb a wide array of social and health factors. In contrast with the sponge-like behavior of SRH among women, SRH among men is thought to be a function of CMCs and multimorbidity alone, uninfluenced by other social and health factors [41].

## 2. Methods

### 2.1. Design and Setting

The design was a cross-sectional survey of economically disadvantaged AA older adults in South Los Angeles. The study was performed between 2015 and 2018 [72,73]. 

### 2.2. Institutional Review Board (IRB)

The study protocol was approved by the Institutional Review Board (IRB) of the Charles R. Drew University of Medicine and Science (CDU), Los Angeles. All participants signed a written informed consent before being enrolled in the study. Participants received financial incentives.

### 2.3. Process and Data Collection

The data collection included structured face-to-face interviews and a comprehensive assessment of medications. The interviewers collected data on demographic factors (age and gender), SES (educational attainment, financial difficulty), objective health (CMCs), and subjective health (SRH and depression).

### 2.4. Participants

The study recruited a convenience sample of economically disadvantaged AA older adults from low-income areas in South Los Angeles, such as the Watts area. Using a convenience sample, AA older adults were eligible if they were AA, were 55 years or older, could complete an interview in English, and resided in the Service Planning Area (SPA) 6. Institutionalized participants were excluded from the study. Other exclusion criteria included being enrolled in any other clinical trials or having poor cognitive performance. This sampling resulted in 740 AAs aged 55 years and older. Our participants were recruited from eleven senior housing apartment units, sixteen predominantly AA churches, and several low-income public housing projects, all located in SPA 6 of Los Angeles County. All of our participants were low-income, underserved, older AAs. The vast majority of older adults in SPA 6 are AAs (49%). About 28% of SPA 6 households are below the federal poverty line (FPL) and 58% of adults have income levels less than 200% of the FPL. About 36% of adults in SPA 6 are uninsured. Between 2013 and 2015, the percentage of homeless AAs in SPA 6 has almost doubled from 39% to 70% [72,73].

### 2.5. Measures

The current study collected data on demographic factors (gender and age), SES (educational attainment and marital status), and health status (multimorbidity, depression, and SRH). 

#### 2.5.1. Dependent Variable

*Self-rated health.* We asked participants about their overall health. The responses ranged from excellent (1) to poor (5). We treated SRH as a continuous variable with a range from 1 to 5, where a higher score reflects worse health. Poor SRH predicts all-cause mortality in the general population [10,74,75,76] as well as in patients with chronic disease [77,78]. Review articles and multiple original studies have established the high predictive validity of poor SRH as a robust determinant of mortality risk, above and beyond confounders such as SES and health [4,10,79].

#### 2.5.2. Mediator

*Depression.* This study used the 15-item Geriatric Depression Scale (GDS) Short Form [80] to evaluate depression. Possible responses were “yes” or “no.” A summary score was calculated with a potential range between 0 and 15. A higher score indicated more depression. The GDS Short Form is a highly reliable and valid instrument that has been used extensively in both clinical and community settings to measure depression among older adults [80].

#### 2.5.3. Independent Variable

*Multimorbidity/number of chronic medical conditions (CMCs).* In this study, multimorbidity was defined as the number of CMCs. Participants were asked about the presence of 11 CMCs. Individuals were asked by the interviewer if a physician had ever told them that they have any of these conditions: Hypertension, heart disease, diabetes, lipid disorder/hypercholesterolemia, cancer, asthma, osteoarthritis, thyroid disorder, chronic obstructive pulmonary disease, rheumatoid arthritis, or gastrointestinal disease [42,81]. Self-reports provide valid information regarding CMCs, although some bias in this approach is to be expected [39,82,83,84,85,86,87]. 

#### 2.5.4. Confounders

*Sociodemographic covariates.* Age, educational attainment, and marital status were the covariates in this study. Age was treated as an interval variable. Educational attainment was operationalized as an interval variable (years of schooling). Higher scores indicated more years of education. Marital status was a dichotomous variable (1 = married, 0 = unmarried)

#### 2.5.5. Moderator

*Gender.* Gender was the effect modifier. Gender was treated as a dichotomous variable (1 = female, 0 = male).

### 2.6. Statistical Analysis

SPSS 22.0 (SPSS Inc., Chicago, IL, USA) and AMOS 22.0 were used to conduct the data analysis. The frequency (%) and the mean (SD) were reported to describe the sample at the baseline and 10 years later. Pearson correlation was used to calculate the bivariate correlations in the overall sample.

A multi-group Structural Equation Model (SEM) was used for multivariable analysis [54]. In our models, groups were defined based on gender. The number of CMCs (multimorbidity) was the predictor, SRH was the outcome, depression was the mediator, and age, education, and marital status were covariates. These variables were selected based on a review of the literature and on the available variables in our data set. The study did not collect data on income; however, most participants were low income AAs and all lived in economically disadvantaged areas of LA County. We did not include health insurance in our analysis because almost all of our participants had health insurance (mostly Medicare or MediCal). To handle missing data, the Full Information Maximum Likelihood (FIML) method was used. Data were missing in less than 1% of the cases. The final SEM model did not include any constraints or co-variances for errors. The model’s goodness-of-fit was assessed using conventional methods: A non-significant chi-square test (*p* > 0.05), a comparative fit index (CFI) larger than 0.95, a root mean squared error of approximation (RMSEA) of less than 0.06, and an X2 with less than 4 degrees of freedom. We reported unstandardized regression coefficients for each path.

## 3. Results

### 3.1. Descriptive Statistics

A total number of 740 AA economically disadvantaged older adults 55 years or older were enrolled in this study, of which 266 were AA men and 474 were AA women. Table 1 describes the sample, both pooled and by gender. This table shows that AA men and AA women differed in age, number of CMCs, depression, and SRH. 

### 3.2. Bivariate Correlations

Table 2 shows the correlation matrix between all the study variables of the sample, both pooled and by gender. As this table shows, in the pooled sample, number of CMCs, depression, and SRH were correlated in the pooled sample and AA men and AA women.

### 3.3. Structural Equation Modeling (SEM) in the Pooled Sample

**Figure 1:**
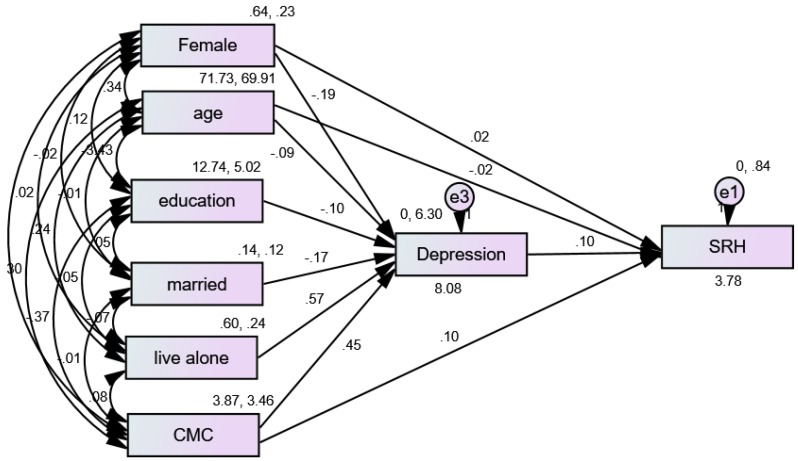
Summary of the Structural Equation Modeling (SEM) overall.

The fit of our first model was very good (CMIN = 4.11, degree of freedom [DF] = 3, *p* = 0.250, CMIN/DF = 1.370, CFI = 0.998, RMSEA = 0.022 (90%CI = 0.000–0.070). Figure 1 shows the results of a SEM with multimorbidity (number of CMCs) as the predictor, depression as the mediator, and SRH as the outcome variable in the pooled sample. According to this model, depression only partially mediated the effects of multimorbidity on SRH in the pooled sample (Table 3). 

### 3.4. Structural Equation Modeling (SEM) in African American (AA) Men

**Figure 2:**
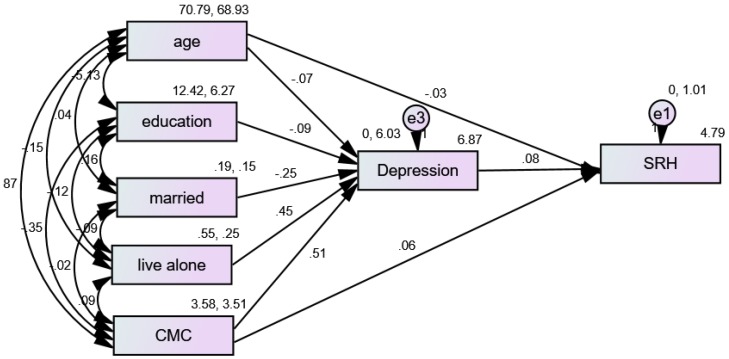
Summary of the Structural Equation Modeling (SEM) in African American (AA) men.

The fit of our multi-group model was very good (CMIN = 5.22, DF = 6, *p* = 0.001, CMIN/DF = 8.981, CFI = 1.000, RMSEA = 0.000 (90%CI = 0.000–0.044). Figure 2 shows the results of an SEM with number of CMCs (multimorbidity) as the predictor, depression as the mediator, and SRH as the outcome variable for AA men. According to this model, depression fully mediated the effects of multimorbidity on SRH in the pooled sample. In a model that also included depression (as the mediator), multimorbidity did not impact SRH for AA men (Table 4).

### 3.5. Structural Equation Modeling (SEM) in African American (AA) Women

**Figure 3:**
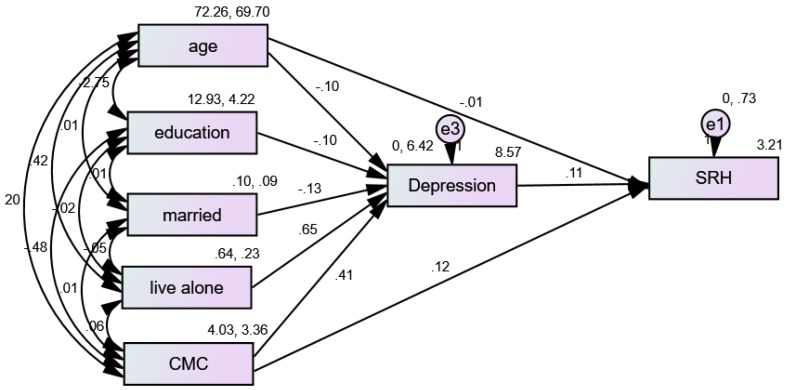
Summary of the Structural Equation Modeling (SEM) in African American (AA) women.

Figure 3 shows the results of an SEM with multimorbidity as the predictor, depression as the mediator, and SRH as the outcome variable for AA women. According to this model, depression only partially mediated the effects of multimorbidity on SRH in the pooled sample. While depression was in the model, multimorbidity still impacted SRH for AA men (Table 4).

## 4. Discussion

In this convenience sample of economically disadvantaged AA older adults, there were gender differences in the way depression mediated the association between multimorbidity and SRH. Depression fully mediated the association between multimorbidity and poor SRH in economically disadvantaged AA men, but not in economically disadvantaged AA women. Depression was the reason low-income AA men with multimorbidity reported poor SRH, but it was more than depression that caused low-income AA women with multimorbidity to report poor SRH.

In a recent study of a smaller sample of low-income AA adults, the SRH of women was found to operate like a sponge, absorbing more affective and contextual information, as opposed to AA men’s SRH [83]. However, that study did not differentiate mediators of SRH by gender, as we have done. 

Our results contribute to the literature on gender differences in SRH. In studies conducted in mainly White samples, poor SRH predicted the risk of mortality among men much better than among women [41,88]. In one of the studies, the author argued that in women, SRH may reflect more contextual and affective information, whereas for men, the main determinant of SRH is multimorbidity (number of CMCs) [41]. In another study, gender difference in the predictive power of poor SRH on the risk of mortality was attenuated by controlling for co-morbid conditions, suggesting that multimorbidity is one of the reasons SRH better predicts mortality among men than among women [88]. However, most of this research used samples that were predominantly White [41]. The main contribution of this study is to extend this literature to AAs. In a study of AA individuals with diabetes, SRH reflected glucose control for AA men but not for AA women [67]. In another study of people with diabetes, worse glycemic control (higher HbA1c) was associated with worse levels of SRH in males and females only when all age groups were combined. However, in younger people, the same association was stronger for women than for men, probably due to diabetes-related worries as a result of high HbA1c [89]. 

In contrast to our results, there are also studies that do not confirm major gender differences in SRH. In one study that spanned 12 years, the Health and Retirement Study (HRS), males and females were compared for trajectories and determinants of SRH. The study, which is mainly composed of Whites, did not show gender differences in SRH levels at baseline. However, SRH declined faster for men than for women over time. Onset of development of CMCs, health behaviors such as smoking, and rate of retirement explain this gender difference in trajectory of SRH over time [90]. In a study that used the 2002–2015 National Health Interview Survey (NHIS) data, ordered logistic regression models were applied to predict SRH as a function of two dozen health conditions, including multimorbidity, physical symptoms, mental health, function, healthcare use, and health behaviors, by gender. The study found almost no evidence supporting the sponge hypothesis. The study failed to show systematic gender variation in the structure of SRH. The study showed that men and women use a wide-range of health-related frames of reference, mostly in a similar way, to make judgments regarding their own health. The following gender difference was observed: At mid-life and older ages, men are more likely than women to weigh physical functioning and negative health behaviors as a factor contributing to their SRH. This study suggested that women report worse SRH than men only through mid-adulthood. This pattern reverses as they age. The study also showed that the female disadvantage in SRH is fully attributable to SES differences. The authors argued that SRH can be used to measure gender differences in health [45]. A study of veterans also did not find major gender differences; however, it did find that exposure to warfare casualties was more predictive of SRH for men than women [91]. These results, however, differ from our study, which suggests SRH may not be comparable between AA men and women.

Our study supports the findings of most researchers that race/ethnicity, gender, and SES have complex effects in shaping what poor SRH means [68,81,92,93,94]. For example, education and income improve the SRH of White but not AA individuals and families [63,64,65]. At the same time, SRH predicts risk of mortality of Whites but not AAs [1,56]. This is because SRH does not reflect the same aspect of health for ethnic groups [43,44] and also across countries [42,95,96,97]. In the Fragile Families and Child Well-Being Study, which followed 2407 AAs and 894 Whites for five years for changes in SRH, in all ethnic groups, anxiety and drinking problems were predictive of poor SRH at baseline and over time. The study documented cross-ethnic variation in the combined (additive) effects of anxiety and depression on SRH. For AAs, depression and anxiety both predicted a worse trajectory of SRH over time. For Whites, depression predicted worse baseline SRH, while anxiety predicted better SRH at baseline and over time [92]. In another cross-sectional study, which borrowed data from the National Survey of American Life 2003 and included 3570 AAs, anxiety and depression had independent (i.e., separate) effects on mental SRH [68]. 

Our results suggest that AA men demonstrate lower SRH when depressed compared to AA women. This is an interesting finding that highlights a relative disadvantage of AA men compared to AA women when it comes to the impact of depression on SRH in the presence of multimorbidity. This finding contributes to the literature on race, gender, and health. The older work of James Stewart [98] and the more recent work of Tommy Curry (*The Man-Not*) [99] help us understand the contribution of structural racism in the life of AA men. Studies by Watkin [100,101], Powell [102,103,104], Neighbors [105], and Griffith [106] show us the interpersonal aspects of depression among AA men. Their work helps us understand the multi-level determinants of depression in AA men, suggesting that a combination of masculinity and racism increases the risk of depression for AA men. This is probably why even among high SES AA men, but not among high SES AA women, we observe an increased risk of depression and psychological distress [81,107,108].

One of the findings of this study was that AA women may be more vulnerable to the effects of living alone on depression compared to AA men. While social support is shown to be important for mental health in all groups [109,110,111], social relations are particularly consequential for AAs [112,113,114,115,116]. Social support promotes mental health directly and buffers the effect of stress [117]. There is literature that suggests social support may be more crucial for mental health of AAs than Whites [118,119,120,121]. In these studies, social support shows a stronger effect on the mental health of AAs than Whites. There are also many studies showing different relevance of social support to the health and wellbeing of men and women [70,122,123,124,125,126,127,128,129,130,131,132,133,134].

### Limitations

There are several limitations to this study, which may be inherent to our study design. Due to a cross-sectional study design, we cannot infer causal associations. We also did not have data on personal or household income. We did not expect a large distribution of income, as all participants were of retirement age and were living in one of the most economically disadvantaged areas of South LA. Health insurance was also present in almost all our participants. Finally, we did not include marital status to reduce collinearity, because we had living alone as a confounder. Furthermore, self-reporting bias must be recognized as a possibility since we did not have access to clinical validations of CMCs or formal diagnoses by mental health providers. There is a need for future studies to replicate these findings using medical chart review or administrative data. The smaller number of AA men in comparison with AA women in our sample may have resulted in differential statistical power. Finally, non-random sampling reduces the generalizability of our results. These limitations were inevitable because we performed a secondary analysis of an existing data set. Despite these limitations, this study contributes to the literature on the intersections of race, gender, and the meaning of SRH, with a particular focus on older adults in a low-income urban setting. 

## 5. Conclusions

In summary, there are gender differences in SRH among low-income AA older men and women with multimorbidity. For low-income AA older men, depression is the reason individuals with multimorbidity report poor SRH. For low-income AA older women, more than depression is involved. More research is needed to investigate other factors contributing to poor SRH among AAs with multimorbidity. Future research should examine whether pain, anxiety, social isolation, or other domains have an impact on SRH among low-income AA women with multiple CMCs.

## Figures and Tables

**Table 1:** Descriptive Statistics of the sample, both pooled and by gender.

	All *n* = 740		African American Men *n* = 262		African American Women *n* = 474	
	Mean	SD	Mean	SD	Mean	SD
Age *	71.73	8.37	70.79	8.32	72.26	8.36
Educational Attainment *	12.74	2.24	12.42	2.51	12.93	2.06
Number of CMCs (Multimorbidity) *	3.86	1.86	3.58	1.88	4.03	1.83
Depression	2.47	2.77	2.53	2.76	2.43	2.79
Self-Rated Health (SRH)	3.13	1.02	3.12	1.09	3.14	0.97
	***n***	**%**	***n***	**%**	***n***	**%**
Married *						
No	640	86.5	215	80.8	425	89.7
Yes	100	13.5	51	19.2	49	10.3
Living Alone *						
No	294	39.7	121	45.5	173	36.5
Yes	446	60.3	145	54.5	301	63.5

CMC: chronic medical condition; SD: Standard Deviation; * *p* < 0.05 (independent sample *t*-test).

**Table 2:** Bivariate correlation matrix of the sample, both pooled and by gender.

Characteristics	1	2	3	4	5	6	7	8
**All**								
1 Gender (Female)	1	0.08 *	0.11 **	−0.12 **	0.09 *	0.12 **	−0.02	0.01
2 Age			−0.18 **	−0.00	0.06	−0.02	−0.25 **	−0.22 **
3 Education			1	0.06	−0.04	−0.09 *	−0.07	−0.03
4 Married				1	−0.41 **	−0.02	−0.07	−0.08 *
5 Living alone					1	0.09 *	0.12 **	0.08 *
6 Number of CMCs (Multimorbidity)						1	0.32 **	0.27 **
7 Depression							1	0.37 **
8 Self-Rated Health (SRH)								1
**AA Men**								
2 Age		1	−0.25 **	0.01	−0.04	−0.06	−0.23 **	−0.28 **
3 Education			1	0.16 **	−0.10	−0.07	−0.06	−0.04
4 Married				1	−0.48 **	−0.03	−0.10	−0.11
5 Living alone					1	0.10	0.15 *	0.12
6 Number of CMCs (Multimorbidity)						1	0.38 **	0.19 **
7 Depression							1	0.30 **
8 Self-Rated Health (SRH)								
**AA Women**								
2 Age		1	−0.16 **	0.00	0.10 *	−0.01	−0.27 **	−0.19 **
3 Education				0.01	−0.02	−0.12 **	−0.07	−0.03
4 Married				1	−0.35 **	0.02	−0.05	−0.06
5 Living alone					1	0.06	0.11 *	0.05
6 Number of CMCs (Multimorbidity)						1	0.29 **	0.32 **
7 Depression							1	0.41 **
8 Self-Rated Health (SRH)								1

* *p* < 0.05, ** *p* < 0.01.

**Table 3:** Summary of the Structural Equation Modeling (SEM) in the pooled sample.

Characteristics	Estimate (S.E.)	*p*
**→ Depression**		
Gender (female)	−0.19 (0.20)	0.340
Marital status (married)	−0.17 (0.30)	0.559
Number of CMCs (multimorbidity)	0.45 (0.05)	<0.001
Living alone	0.57 (0.21)	0.006
Age	−0.09 (0.01)	<0.001
Education	−0.10 (0.04)	0.022
**→ Self-Rated Health (SRH)**		
Gender (female)	0.02 (0.07)	0.795
Age	−0.02 (0.00)	<0.001
Number of CMCs (multimorbidity)	0.10 (0.02)	<0.001
Depression	0.10 (0.01)	<0.001

SE: Standard Error; CMC: chronic medical condition

**Table 4:** Summary of the Structural Equation Modeling (SEM) in African American (AA) men and women.

Characteristics	Estimate (S.E.)	*p*	Estimate (S.E.)	*p*
	Men		Women	
**→ Depression**				
Marital status (married)	−0.25 (0.44)	0.571	−0.13 (0.41)	0.752
Number of CMCs (multimorbidity)	0.51 (0.08)	<0.001	0.41 (0.06)	<0.001
Living alone	0.45 (0.35)	0.193	0.65 (0.26)	0.012
Age	−0.07 (0.02)	<0.001	−0.10 (0.01)	<0.001
**→ Self-Rated Health (SRH)**				
Education	−0.09 (0.06)	0.162	−0.10 (0.06)	0.078
Age	−0.03 (0.01)	<0.001	−0.01 (0.01)	0.021
Number of CMCs (multimorbidity)	0.06 (0.04)	0.115	0.12 (0.02)	<0.001
Depression	0.09 (0.03)	<0.001	0.11 (0.02)	<0.001

SE: Standard Error; CMC: chronic medical condition
